# A mathematical model to estimate the seasonal change in apparent longevity of bee colony

**DOI:** 10.1038/s41598-019-40725-0

**Published:** 2019-03-11

**Authors:** Yasuhiro Yamada, Toshiro Yamada, Kazuko Yamada

**Affiliations:** 1 Department of Applied Physics, Graduate School of Engineering, University of Tokyo, Hongo 7-3-1, Bunkyo-ku, Tokyo, 113-8656 Japan; 2 Graduate School of Natural Science & Technology, Kanazawa University, Kakuma-machi, Kanazawa, 920-1192 Japan; 3 Present Address: Department of Physics, Osaka University, 1-1 Machikaneyama, Toyonaka, Osaka, 560-0043 Japan; 4 Present Address: 2-10-15, Teraji, Kanazawa, Ishikawa, 921-8178 Japan

## Abstract

The longevity of a honeybee colony is far more significant than the lifespan of an individual honeybee, a social insect. The longevity of a honeybee colony is integral to the fate of the colony. We have proposed a new mathematical model to estimate the apparent longevity defined in the upper limit of an integral equation. The apparent longevity can be determined only from the numbers of adult bees and capped brood. By applying the mathematical model to a honeybee colony in Japan, seasonal changes in apparent longevity were estimated in three long-term field experiments. Three apparent longevities showed very similar season-changes to one another, increasing from early autumn, reaching a maximum at the end of overwintering and falling approximately plumb down after overwintering. The influence of measurement errors in the numbers of adult bees and capped brood on the apparent longevity was investigated.

## Introduction

A lifespan of an animal, which is the period of time while an individual is alive, is an important index to evaluate individual activities. In the colony composed of eusocial insects such as honeybees (*Apis mellifera*) which exhibit age-polyethism, the lifespan of each individual cannot always give an assessment as to the activities of a colony but the longevity of colony could give it more appropriately. The longevity of a colony will have greater significance than the lifespan of each individual of the colony. The life of colony diversely depends on the inborn lifespan of an individual, the labor division distribution ratio of each honeybee performing a particular duty, the natural environment such as the weather, the amount of food, pests and pathogens, the environmental pollution due to pesticides and so on.

The honeybee length of life has been observed or estimated before in the four seasons, which have a distinct bimodal distribution in temperature zones. According to previous papers, honeybees live for 2–4 weeks^1^ and 30–40 days^2^ in spring, for 1–2 weeks^1^, 25–30 days^2^ and 15–38 days^3^ in summer, for 2–4 weeks^1^ and 50–60 days^2^ in autumn, and for 150–200 days^3^, 253 days^2^, 270 days^4^, 304 days^5^ 6–8 months^6^ and 150–200 days^3^ in winter, where it has been estimated that the difference of life length among seasons may come from the brood-rearing load imposed on honeybees^1^ and may mainly come from foraging and brood-rearing activity^2^. Incidentally, the lifetime of the queen seems to be three to four years (maximum observed nine years). The average length of life of worker bees in laboratory cages was observed to range from 30.5 to 45.5 days^7^. The study on the influence of altitude on the lifespan of the honeybee has found that the lifespans are 138 days at an altitude of 970 m and 73 days at an altitude of 200 m, respectively^8^.

Many papers have discussed what factors affect the length of life (lifespan, longevity, life expectancy) on a honeybee colony as follows:

Proper nutrition may increase the length of life in a honeybee colony. Honeybees taking beebread or diets with date palm pollen (the best source for hypopharyngeal gland development) showed the longest fifty percent lethal time (LT_50_)^9^. The examination for the effect of various fat proteins on honeybee longevity have shown that honeybees fed diets of red gum pollen have the longest lifespan but those fed invert sugar have the shortest lifespan^10^. In the discussion on nutrition-related risks to honey bee colonies such as starvation, monoculture, genetically modified crops and pesticides in pollen and sugar, protein nutrient strongly affects brood production and larval starvation (alone and or in combination with other stresses) can weaken colonies^11^. And protein content in larval diet could positively affect worker longevity likely because of the increased antioxidant gene expression^12^. Through study on the effect of different dietary sugars and honey on longevity in hyper parasitoids, it has been found that reproductive success and longevity in honeybees varied significantly when fed different sugars^13^. It has been demonstrated that resveratrol significantly affects gustatory responsiveness and prolonged lifespan in wild-type honeybees under normal oxygen conditions, but the enhanced lifespan effect of resveratrol is abolished under hyperoxic conditions^14^. Comparisons among the nutritional effects of high protein feeds on honeybees in three steps of incubator, field and overwintering have shown that the longevity of caged honeybees in incubator is significantly affected by dietary treatments^15^. They concluded that soybean flour and bread yeast can be used as pollen supplements and substitute cakes. From the discussions on the ontogenic time and worker longevity in the Australian stingless, it has been inferred that extended longevity may result from evolutionary adaptations to the floral resource scarcity^16^. It has been demonstrated that pollen and royal jelly extend worker longevity^17^.

It is reported that the load of work imposed on honeybees is deeply related to their length of life as follows: It has been developed that workloads imposed on worker bees beyond those normally encountered by them make their lifespans shorter^18^. The higher longevity of workers in winter seems to be probably connected with the interruption of the brood-rearing cycle^1^. Foraging and brood rearing activities are regarded as important factors affecting the lifespan^2^. Based on the hypothesis that the division of labor in foraging workers is a consequence of division of risk among foragers with differing life expectancies through field experiment, the validity of a dynamic programming model has been discussed^19^. A reverse relationship between the longevity of honeybees has been reported^20^. On the main driver of aging in long-lived winter honeybees, it has been concluded that all processes driving senescence in honeybees will have to be considered separately not only between queens and workers but also within the worker caste^21^. It has been inferred from their experiments that the mortality patterns in worker honeybees mainly influenced by the transition from in-hive workers to foragers, where the nearly tenfold lifespan differences between winter and summer bees defies explanation by the fact that the reduction of foraging activity and elimination of a number of external mortality factors increases the life expectancy of workers only by 4–5 days though it was experimentally confirmed that there was a higher mortality rate of foragers than non-foraging bees^22^. Furthermore, it was found that honeybees live longer in smaller than in larger colonies and it was concluded that it was caused by the primary effect of simultaneous consequences of foraging choice, overall workload and the rate of aging of worker honeybees^23^.

The influence of a physical environment on the length of life in a honeybee colony is discussed. Comparing the longevity between field colonies and laboratory caged ones, there are seasonal inconsistencies between the two^24^. It has also been reported that eye color of honeybees can cause differences in longevity^25^.

The influence of reproductive protein (vitellogenin) and brood (juvenile) pheromone on longevity has been discussed. The role of the vitellogenin in honeybee workers has been reviewed and a mathematical model has been proposed^6^. Vitellogenin gene activity protects worker bees from oxidative stress, where vitellogenin protein is synthesized at high levels in honeybee queens and is abundant in long-lived workers^26^. It has been demonstrated that brood rearing reduces worker vitellogenin stores and colony long-term survival^27^. It has been discussed how the manipulation of social environment in colonies with long-lived winter bees might alter the pace of functional senescence as well as of cellular senescence which is induced by such social intervention as changes in social task behaviors and social environment^28^.

Extrinsic influences such as mites, pathogens and pesticides on the longevity have been examined. The examination of sub-lethal effects, including delayed larval development and adult emergence or shortened adult longevity on worker honey bees from pesticide residue exposure from contaminated brood comb, shows they can have indirect effects on the colony such as premature shifts in hive roles and foraging activity^29^. *Nosema ceranae* can infect honeybee larvae, reducing subsequent adult longevity^30^.

Based on the information from observations of life length and probable factors influencing it as mentioned above, several mathematical models have been proposed in order to estimate seasonal changes in the length of life as follows:

An attempt has been made to estimate the length of life in a honeybee colony assuming life expectancy as a function of both the future hatching rates and the net changes of the worker bee populations based on the number of worker-bees and open and sealed brood-cells observed at regular intervals^31^. A mathematical model with arbitrary parameters has been proposed under some assumptions to estimate the relationship between worker longevity and the intracolonial population dynamics of honeybees in temperature zones from early spring to late autumn^32^. And two mathematical models with arbitrary parameters have been proposed to estimate expected longevity of workers in colonies with age polyethism and that with caste polyethism under several assumptions while comparing that without each polyethism^33,34^. Under several assumptions in order to offer a simple theoretical framework with which to explore how the dynamics of food flow through a colony might interact with population dynamics to determine colony growth, a mathematical model with arbitrary parameters has been proposed^35,36^. Honeybee populations are predicted by using several parameters such as mortality within each bee caste under some assumptions^37^.

These mathematical models might predict correctly the length of life and be useful and effective in beekeeping if the values of arbitrary parameters and the assumptions are close to the truth. The more parameters in a model, the more information can be obtained. The degree of accuracy in estimation deeply depends upon the veracity of each parameters value and the validity of each assumption. A single inaccurate parameter or assumption may lead to a large error in simulated results. It is difficult to correctly estimate the errors arising from the arbitrary parameters and the assumptions.

The length of life in a honeybee colony is one of the most important indexes to assess the colony activity. Here we call the length of life in a honeybee colony the “apparent” longevity which reflects the influences of environment due to the weather and harmful pollutants such as pesticides, those of infections by pathogens, mites, wax-moth larvae and small hive-beetles, those of predation by birds, hornets and toads, and those of missing bees which are alive elsewhere. This work is focused on a proposal of a mathematical model to estimate only the apparent longevity, *L(t)*, fairly accurately through all seasons. In order to estimate the apparent longevity as accurately as possible, the less parameters and the more plausible assumptions are better and it is desirable that the parameters can be determined from experimental data with high accuracy and the assumptions are closer to the truth. In this paper we propose a simple and realistic mathematical model whose parameters can be determined from the numbers of adult bees and capped brood obtained from the photos of combs and the inside of a beehive, which are comparatively easy to be accurately obtained from an experiment.

## Examples of applying the model

**Figure 1:**
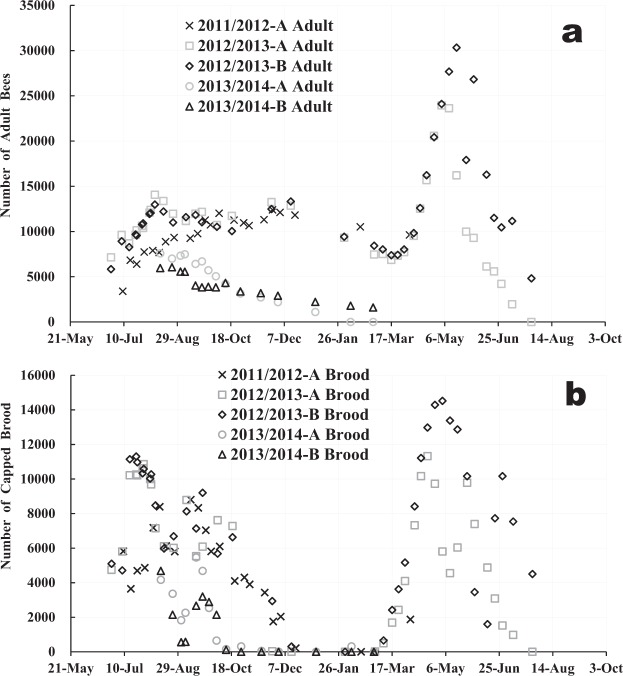
Seasonal change in the numbers of adult bees (**a**) and capped brood (**b**) in three long-term field experiments. The field experiments to obtain the numbers of adult bees capped brood were conducted as carefully as possible because their measurement accuracy could greatly affects the estimation accuracy of apparent longevity. Their numbers accurately counted with the help of an newly-developed image processing software while being directly corrected on enlarged photographs of all combs and the inside of a hive because their numbers obtained from photographs could be counted again at any time when in doubt. An experiment is conducted just after dawn before foraging bees go out anywhere.

We obtained the numbers of adult bees and capped brood from three long-term field experiments that we conducted in 2011/2012 from July 2011 to April 2012^38^, 2012/2013 from June 2012 to July 2013^39^ and 2013/2014 from August 2013 to February 2014^40^ in the same apiary in Shika, Japan as shown in Fig. 1 and Supplementary Table 1. To apply the proposed model to honeybee colonies, we made the following assumptions: (1) A pupa (brood) in a sealed (capped) cell is removed as soon as the pupa dies. (2) At the start of the experiment (when t = t_0_), the age distribution of the initial capped brood is uniform (i.e., the number of initial capped brood at each age (in days) is equal to one-twelfth the total initial number). (3) The eclosion rate, *p(t)*, has a constant value of 0.9^41^ throughout all seasons, although it may possibly be affected by other factors such as the ratio between the number of in-hive bees and foraging bees. (4) After eclosion, adult bees unexpectedly disappear (except in case of death through capture by birds, frogs, hornets, dragonflies, etc.) and cannot return to their beehive because of sudden changes in the weather and other factors such as *in situ* death due to pesticides and the onset of darkness. Here, apparent longevity includes all environmental factors, except the eclosion rate.

**Figure 2:**
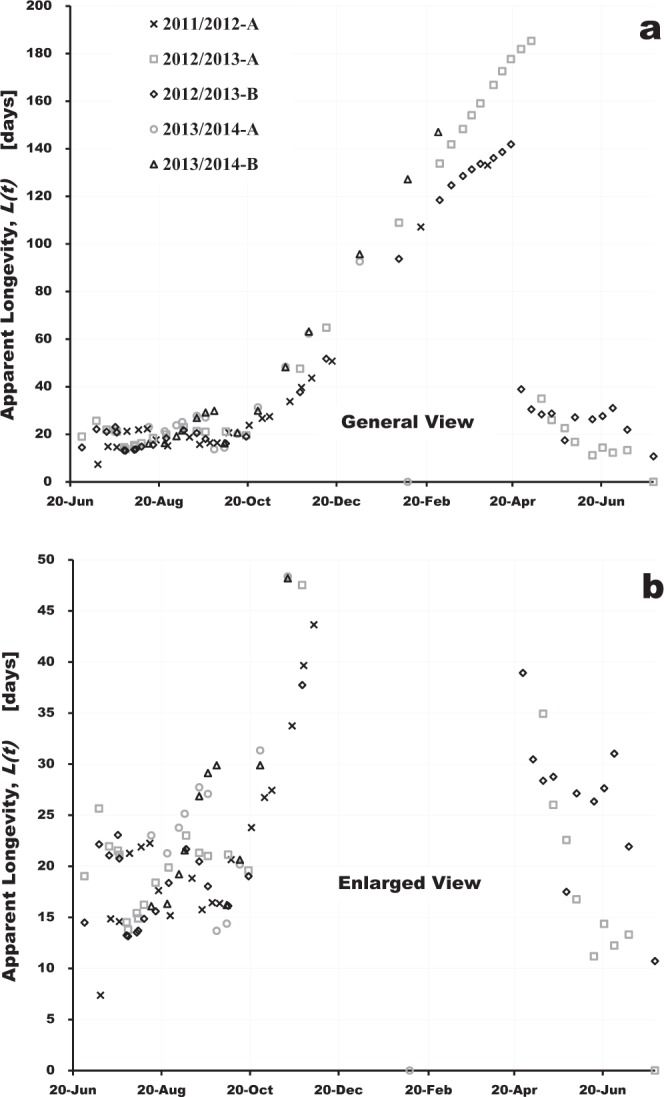
Seasonal change in apparent longevity at a general view (**a**) and that at an enlarged view (**b**).

From the abovementioned assumptions, we have estimated the apparent longevity of a honeybee colony using the numbers of adult bees and capped brood in Fig. 1 and Supplementary Table 2. Figure 2 shows the change in apparent longevity for each colony with days that have elapsed from June 28 in the 2011/2012^38^, 2012/2013^39^ and 2013/2014^40^ experiments. We can perceive from Figs 1 and 2 that each estimated apparent longevity roughly has a similar change with the elapsed days regardless of different initial conditions and different numbers of adult bees and capped brood in each colony as follows: The apparent longevity begins to increase from late September and continues to increase until spring and after that it decreases suddenly in mid spring. That the longevity involving a sudden change was easily obtained from the measured numbers of adult bees and capped brood by solving a set of equations suggests from a mathematical viewpoint that both measured data were considerably accurate and little inconsistent with each other. In order to ensure the validity of the mathematical model, we tried to compare the previous reports^2,31^. The previous results, however, seemed to be insufficient for the validity check (see Supplementary Fig. 1).

Examining the seasonal changes in atmospheric temperature in the Noto district around the experimental site (Weather archives in the Noto district of Ishikawa prefecture in Japan by the Kanazawa Local Meteorological Observatory), those during each experimental period seem to be approximately similar to each other. The similarity of the seasonal changes in apparent longevity of each colony may be due to that in atmospheric temperature during each experimental period. In other words, the apparent longevity in a colony will probably be very susceptible to the weather conditions. Strictly, the seasonal changes in apparent longevity, however, do not always coincide with those in atmospheric temperature. Especially, a drastic drop of apparent longevity in spring is distinct from a gentle rise in atmospheric temperature. This fact implies that the apparent longevity may come under the influence of not only atmospheric temperature but also other factors.

Examining the seasonal changes in apparent longevity carefully, we can generally know the following findings: Almost every colony begins to increase in apparent longevity from late September even before it gets cold, then continues to increase through late April after overwintering and shortly afterward the apparent longevity decreases extremely rapidly. The extreme rapid decrease in apparent longevity in spring cannot be explained only from the environmental conditions which are weather conditions^1^, nutrition^9^, workloads such as foraging and brood-rearing^2,20^, extrinsic factors such as pathogens and pesticides^29^. Consequently, it seem plausible that the information on longevity may be genetically programmed to vary drastically as certain threshold values of environmental conditions such as atmospheric temperature are exceeded. On the other hand, a slight elevation of the apparent longevity shown in September may be caused by less foraging load in August when there are few flowers in bloom

Why was the apparent longevity of the 2012/2013-B colony longer than that of the 2012/2013-A colony by about nine days after the drastic drop? Examining the records such as photographs and our research notes in the 2012/2013 experiment in further detail, we found that several queen cells were made on the combs only in the 2012/2013-B colony after overwintering (May 10, 2013), and afterwards the old queen in the 2012/2013-B colony was replaced by a new queen (May 26, 2013).

From the above fact, we infer as follows: When the queen is lost, eggs which the old queen lays before she is replaced by a new one must survive longer because there is a time lag of oviposition between the old queen and a new one and the time lag may lead to a lack of nurse bees. Examining in more detail, the apparent longevity of the 2012/2013-B colony is maintained at an approximately constant value higher than that of the 2012/2013-A colony from early May to late June in 2013, and then it decreases to about the apparent longevity of the 2012/2013-A colony in early July. It may be inferred from this fact that the old queen continues to lay eggs with higher longevity untill she is replaced by a new queen. When queen cells begin to be made, the new queen begins to lay eggs with normal longevity. This may be genetically programmed.

Comparing the apparent longevities of colonies which had succeeded in overwintering (2012/2013-A and B in Fig. 2), we found that the longer apparent longevity colony (2012/2013-A) during overwintering becomes smaller in size than the other after overwintering against expectations, judging from the numbers of adult bees and capped brood as shown in Fig. 1.

## Effect of miscount errors in measurement

When we obtain the numbers of adult bees and capped brood in a field experiment, the following errors sometimes creep in: (1) Errors arising from a counting method, where a weighing method is apt to have much more errors than a direct counting method: (2) Errors arising from the timing of measurement, where a measurement just after dawn before foraging bees leave from their beehive contains less errors than that in the daylight. We will discuss the effect of miscounting error on the apparent longevity and examine the possibilities that the apparent longevity can be used as a kind of consistent test to evaluate the soundness of experimental data in a field experiment.

To take concrete examples, let us consider four cases where the number of adult bees is overestimated more than the original one of the 2013/2014-A colony by 20% (CASE 1) and it is underestimated less than the original by 20% (CASE 2); the number of capped brood is overestimated more than the original by 20% (CASE 3) and it is under estimated less than the original by 20% (CASE 4) for about one month from August 24, 2013 to September 27, 2013 in the 2013/2014 experiment. These estimated results of apparent longevity are shown in Table 1. From Table 1, we can find that the overestimations of adult bees and the underestimations of capped brood give longer apparent longevity than the original longevity or vice versa, and that a difference in the number of adult bees from the original for a given miscounted period affects the apparent longevity only for the given miscounted period. The difference does not affect the apparent longevity for a correctly counted period when there is no difference in adult bees from the original. A difference in the number of capped brood from the original for a given period continues to affects the apparent longevity even over a long period when there is no difference in the number of capped brood from the original.

**Table 1:** Estimated apparent longevity (*L(t)*) of 2013/2014-A in several cases where adult bees or capped brood is miscounted by plus or minus 20% from August 24 to September 27.

Date	Elapsed day	Calculated *L(t)* [day]				
		Original	CASE 1	CASE 2	CASE 3	CASE 4
13-Aug-13	0	23.02	23.02	23.02	23.02	23.02
24-Aug-13	11	21.26	25.51	17.01	21.26	21.26
1-Sep-13	19	23.77	28.22	19.32	22.54	25.00
5-Sep-13	23	25.14	29.69	20.59	23.21	27.07
15-Sep-13	33	27.73	31.63	23.83	25.01	30.45
21-Sep-13	39	27.08	31.14	21.90	22.65	31.32
27-Sep-13	45	13.68	23.46	10.49	11.04	24.58
4-Oct-13	52	14.39	14.39	14.39	12.48	17.31
13-Oct-13	61	20.18	20.18	20.18	18.71	22.36
27-Oct-13	75	31.34	31.34	31.34	30.33	32.83
15-Nov-13	94	48.37	48.37	48.37	47.27	49.67
1-Dec-13	110	62.34	62.34	62.34	61.58	63.49
5-Jan-14	145	92.61	92.61	92.61	92.33	93.02
7-Feb-14	178	0.00	0.00	0.00	0.00	0.00
28-Feb-14	199	0.00	0.00	0.00	0.00	0.00
Change in number ranging from August 24th to September 27th		Adult bees 0%	Adult bees +20%	Adult bees −20%	Adult bees 0%	Adult bees 0%
		Brood 0%	Brood 0%	Brood 0%	Brood +20%	Brood −20%

[Assumptions in Calculation].

(1) Interval of Capped Broods = 12 days, (2) Emergence Probability (Eclosion rate) from Pupae = 90%.

**Figure 3:**
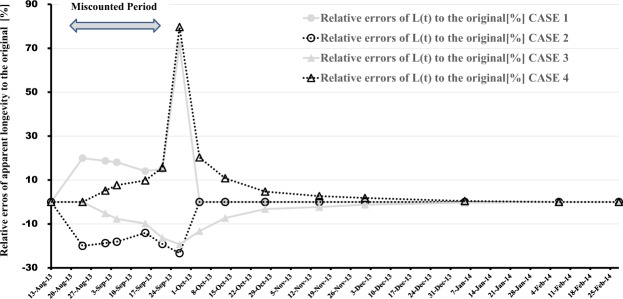
Relative errors of apparent longevity to the original due to measurement errors in four cases.

Figure 3 shows the relative errors of apparent longevity in all cases. It can be seen from Fig. 3 that the maximum value of relative errors in CASE 1 is about plus 72%, that in CASE 2 is about minus 23%, that in CASE 3 is about minus 19% and that in CASE 4 is about plus 80%. The measurement errors in the number of capped brood can affect the apparent longevity over to a long period without the measurement errors though those of adult bees can affect the apparent longevity only for a period with the measurement errors.

## Conclusion

A new mathematical model has been proposed, from which the apparent longevity of honeybee colony can be estimated only from the numbers of adult bees and capped brood. The apparent longevity proposed newly in this paper which is an indicator of overall longevity to characterize a complicated colony system, is likely to provide useful prior information not only on normal environmental conditions of a colony but also on anomalous ones. This mathematical model can be applied to the colonies of any other social insects having a clear life cycle.

Using the mathematical model, we estimated the apparent longevity of a honeybee colony in long-term field experiments. As a result of the estimation, the following were made clear: (1) Similar seasonal changes in apparent longevity can be shown among the three long-term field experiments regardless of the different populations in colonies. (2) A drastic drop in apparent longevity in spring cannot be always explained by conventional theories and is thought to be probably ascribable to programmed code: (3) A slight elevation in apparent longevity in September seems to be caused by less workloads in summer: (4) A difference in apparent longevity of about 9 days after a drastic drop seems to be perhaps induced by the replacement of a queen under conditions of the existence of queen cells. (5) A longer apparent longevity colony during overwintering can lead to a smaller colony after overwintering.

Measurement errors in the number of adult bees or capped brood seem to affect the apparent longevity. For instance, when either the number of adult bees is overestimated or that of capped brood is underestimated, relative errors in apparent longevity increase severalfold. Errors in the number of capped brood have a lasting effect on the apparent longevity compared to a period without measurement errors.

The apparent longevity may be available for checking the consistency of field experimental data in a honeybee colony through the process by which a set of equations to estimate it is solved.

## Methods

### Mathematical model for bee population

Many reports on the population dynamic model of honeybee colony consist of several parameters on environmental conditions. Simulation results depend on the accuracy of these parameters which is difficult to be accurately determined from the vagaries of a variety of environmental conditions. Therefore, we now focus our attention on one parameter model which can assess the anomalous change (bee behavior) in colony due to various environmental factors such as pesticide, temperature, humidity, food, bee-family structure (decay of polyethim) and so on, in order to estimate a long-term change in overall longevity of colony workers.

Based on the numbers of adult bees and capped brood at regular intervals according to the similar procedure reported previously^31^, we attempt to resolve one of the remaining issues in the previous report^31^ such as median age of the colony, mortality rate, median life expectancy, etc. as follows:

It is very important to know the general health status of a honeybee colony in order to predict any anomalous changes such as a massive death and a failure in wintering. General health status can be reflected in the average lifespan of the workers, which is obtained from the ratio of the total stationary population to the number of surviving bees immediately after eclosion. However, a considerable amount of time and energy is required to obtain temporal changes in average worker lifespan in a colony because following up the changes in age distribution of large numbers of individual adult bees is almost impossible. The information which can be easily obtained with high accuracy in a field experiment are the numbers of adult bees and capped brood. The two numbers are highly correlated with each other and also with the duration of life of a honeybee. The maximum duration of life does not always coincide with the longevity in a honeybee colony because the life of a missing honeybee which is alive out of a beehive cannot be considered as the duration of life. Here we will call the maximum duration of life obtained from the two measured numbers an “apparent longevity”. The sound estimation of apparent longevity can be performed by relying on the correct numbers of adult bees and capped brood. The inconsistency between the two measured numbers of adult bees and capped brood can be reflected as a singular apparent longevity which is estimated from the two measured numbers. That is, an apparent longevity (*L*_*a*_) can be expected also as a reliability assessment of the two measured numbers in a field experiment, which will be able to evaluate the soundness of the two measured data obtained from a field experiment as is the case with the check of thermodynamic consistency in vapor-liquid equilibrium data sets^42^. If either or both of the two measured numbers which have a close connection with each other are inaccurate and inconsistent with each other, the apparent longevity cannot be obtained because the following equations cannot be solved. The change in *L*_*a*_ with the passage of time shows the dynamic activity of a honeybee colony which is influenced by the environment (weather, pesticide, enemies of honeybees, etc.).

Here, we will introduce a dynamic parameter called “apparent longevity”, *L*_*a*_, (*L(t)*, defined in Eq. (1)), which characterizes the “border” age at which an average worker bee disappears from the colony:

1 $${\rm{a}}(t)={\int }_{{T}_{pupa}}^{{T}_{pupa}+L(t)}u(t-s)p(t-s)ds,$$

where *a(t)* and *u(t)* denote the number of adult bees and newly capped brood, respectively, per unit time *t*, *p(t)* is the eclosion rate of pupae capped at time *t*, and *T*_*pupa*_ denotes the average capped brood period, which is 12 days. It is noteworthy that if the colony is in a steady state, where the worker bees die at the same age, *L(t)* represents the lifespan of the adult worker bee and may therefore provide an estimation of the longevity of worker bees in a normal colony, where age distribution of dying bees is given by a narrow Gaussian.

To obtain the apparent longevity, *L(t)*, in Eq. (1), it is necessary to know *a(t)*, *u(t)*, and *p(t)*. *a(t)* and *p(t)* may be obtained from our measurements, respectively. Since it is difficult to obtain *u(t)* directly, we estimate it from Eq. (2):

2 $$b(t)={\int }_{0}^{{T}_{pupa}}u(t-s){[p(t-s)]}^{s/{T}_{pupa}}ds,$$

where *b(t)* is the total number of capped brood at time *t*. Note that we obtain Eq. (2) assuming that a pupa is removed immediately when it is dead in a cell before eclosion.

The combination of Eqs (1) and (2) is useful to characterize a colony by using its macroscopic quantities (total numbers of adults and capped brood), which are easily accessible. Though *a(t)* in Eq. (1) and *b(t)* in Eq. (2) are continuous, their measurements which can be obtained every measurement date are discrete in an actual experiment. Therefore, it is necessary to discretize Eqs (1) and (2) in order to obtain the apparent longevity *L(t)*. That is, we obtain sets of data at several time points in an actual experiment rather than continuously. Their time-discretization can be achieved under the assumptions that the number of newly capped brood and the eclosion rate are constant between two successive measurements [i.e., *u(t)* and *p(t)* have the following functional forms]:

3 $$u(t)=v(t-{T}_{egg})=\{\begin{array}{c}\,{\bar{u}}_{0}(t < {t}_{0})\,betore\,starting\,the\,experiment\\ {\bar{u}}_{1}({t}_{0}\le t < {t}_{1})\,from\,the\,initial\,observation\,to\,the\,first\,one\\ {\overline{u}}_{2}({t}_{1}\le t < {t}_{2})\,from\,the\,first\,observation\,to\,the\,second\,one\,\\ \vdots \\ {\overline{u}}_{k}({t}_{k-1}\le t < {t}_{k})\,from\,the\,{(k-1)}^{th}\,observation\,to\,the\,{k}^{th}\,one\\ \vdots \\ {\overline{u}}_{N-1}({t}_{N-2}\le t < {t}_{N-1})\,from\,the\,previous\,observation\,to\,the\,final\,one\end{array}$$

4 $$p(t)=\{\,\begin{array}{c}{\bar{p}}_{0}(t < {t}_{0})\\ {\bar{p}}_{1}({t}_{0}\le t < {t}_{1})\\ {\bar{p}}_{2}({t}_{1}\le t < {t}_{2})\\ \vdots \\ {\overline{p}}_{k}({t}_{k-1}\le t < {t}_{k})\\ \vdots \\ {\overline{p}}_{N-1}({t}_{N-2}\le t < {t}_{N-1})\end{array}$$

where *t*_*k*_ denotes the time at the *k*^*th*^ measurement. The subscript *k* takes from 0 (at initial experiment) to *N − 1* (at final measurement).

Since ${\bar{p}}_{k}$ is given by the eclosion rate reported previously^41^, there are *N*-variables to be determined in Eq. (2): ${\bar{u}}_{0}$,${\overline{u}}_{2}$ …${\overline{u}}_{N-1}$. Using the measured number of capped brood at *t* = *t*_*k*_, *b*(*t*_*k*_), we obtain the following system of linear equations:

5 $$b(tk)={\int }_{0}^{Tpupa}u({t}_{k}-s){[p({t}_{k}-s)]}^{s{/}_{Tpupa}}ds\,for\,k=0,\,\ldots ,\,N-1$$

This system of linear equations is easily solved using standard Gaussian elimination, which yields a set of solutions, ${\bar{u}}_{0}$,${\overline{u}}_{2}$ …${\overline{u}}_{N-1}$. Then, *L(t*_0_), …, *L(t*_N_*)* are found from the discretized version of Eq. (1) by using an iterative procedure, with the values of ${\bar{u}}_{0}$,${\overline{u}}_{2}$ …${\overline{u}}_{N-1}$ obtained from Eq. (5).

Here we will explain the symbols used in this mathematical model below:

*T*_*egg*_ is the egg and larval period (from the time the queen lays eggs till they become a capped brood) and *T*_*egg*_ = 9 days for a honeybee; *T*_*pupa*_ is the capped brood period, mainly pupal period (from the time a brood starts to be capped till the capped brood ecloses); *T*_*pupa*_ = 12 days for a honeybee; *a(t)* is the number of adult bees; *v(t)* is the number of eggs laid by a queen per day in an interval between the *(k − 1)*^*th*^ measurement and the *k*^*th*^; *b(t)* is the total number of capped brood summed up the residual and newly capped brood; *u(t)* is the number of newly capped brood per day in an interval between the *(k − 1)*^*th*^ measurement and the *k*^*th*^ at the elapsed days of *t*, where *u(t)* = *v(t* − *9)*, because it takes 9 days for capping of the honeybee eggs; *t*_*k*_ = time at the *k*^*th*^ measurement, which is a discrete variable, *t* = *t*_0_, *t*_1_, ····, *t*_*N − 1*_, *t*_*N*_; *ū*_*k*_ is equal to *u(t*_*k*_) which is assumed to be constant between the *(k − 1*)^*th*^ measurement and the *k*^*th*^, to obtain the value of *u(t)*, we assume the following because of the fact that the capped brood hatches over 12 days for a honeybee; *L(t)* is the apparent longevity of a colony, which is a parameter to characterize the degree of longevity of that colony. As there are few lost bees in winter because of extremely low foraging activity, the apparent longevity is roughly equivalent to the longevity of oldest member of that group. On the other hand, it is not so always other than in winter; *p(t)* is the probability of the brood newly capped at time *t* eclosing and developing into imagos (adult bees); ${\bar{p}}_{k}$is *p(t*_*k*_*)* that is assumed to be constant between the *(k − 1)*^*th*^ measurement and the *k*^*th*^, which is estimated from the eclosion rate curve of the capped brood.

We assume the following assumptions to solve the above equations: An equal number of brood (larvae) are capped per day between a certain experiment and the next experiment. One-twelfth of all capped brood, *b(k)*, which are measured in the *k*^*th*^ experiment will turned into new adult bees per day between the *k*^*th*^ experiment and *k* + *1*^*th*^ experiment because of the fact that new adult bees emerge from capped brood after 12 days from the day when a brood is capped. When an interval between a certain experiment and the next one is longer than 12 days, the number of new adult bees emerging from capped brood remains the same to the next experiment. In this work Ruby programing language was used to solve the equations in the mathematical model mentioned above. Apparent longevity of a colony composed of social insects other than honeybees can be estimated using the mathematical model proposed in this paper when the life cycle of the insect is known (*T*_*egg*_, *T*_*pupa*_ and *p(t)* can be obtained) and the numbers of imagos and pupae can be accurately measured at given time intervals.

## Supplementary information

A mathematical model to estimate the seasonal change in apparent longevity of bee colony
