# Supplementary material for: A mathematical model to estimate the seasonal change in apparent longevity of bee colony
Source: Sci Rep. 2019 Mar 11;9:4102. doi: 10.1038/s41598-019-40725-0 (PMC6411913; doi:10.1038/s41598-019-40725-0)

A mathematical model to estimate the seasonal change in apparent longevity of bee colony

Yasuhiro Yamada[1†], Toshiro Yamada[2††*], Kazuko Yamada[2††]

[1] Department of Applied Physics, Graduate School of Engineering, University of Tokyo, Hongo 7-3-1, Bunkyo-ku, Tokyo 113-8656, Japan

[2] Graduate School of Natural Science & Technology, Kanazawa University, Kakuma-machi, Kanazawa 920-1192, Japan

†Present address: Department of Physics, Osaka University, 1-1 Machikaneyama, Toyonaka, Osaka, 560-0043, Japan

†† Present address: 2-10-15, Teraji, Kanazawa, Ishikawa, 921-8178, Japan

*Corresponding author. Email: tyamada@staff.kanazawa-u.ac.jp. Present situation: professor emeritus of Kanazawa University

Supplementary Table 1. The numbers of adult bees and capped brood in 2011/2012, 2012/2013 and 2013/2014 experiments. The detailed experimental methods are described in our previous papers[38-40].

Date	Elapsed Days	2011/2012 EXP-2011-A	
		Adult	Brood
9-Jul-11	0	3392	5819
16-Jul-11	7	6827	3644
22-Jul-11	13	6399	4692
29-Jul-11	20	7737	4861
6-Aug-11	28	7893	7179
12-Aug-11	34	7675	8390
18-Aug-11	40	8873	6125
26-Aug-11	48	9327	5797
10-Sep-11	63	9249	8803
17-Sep-11	70	9762	8327
24-Sep-11	77	11252	7034
29-Sep-11	82	10736	5810
7-Oct-11	90	12015	6100
21-Oct-11	104	11253	4100
30-Oct-11	113	10958	4300
4-Nov-11	118	10654	3900
18-Nov-11	132	11303	3430
26-Nov-11	140	12390	1750
3-Dec-11	148	12109	2038
17-Dec-11	162	11811	212
16-Feb-12	223	10514	0
2-Apr-12	269	9622	1873

Date	Elapsed Days	2012/2013 EXP-2012-A		2012/2013 EXP-2012-B	
		Adult	Brood	Adult	Brood
28-Jun-12	0	7136	4746	5832	5094
08-Jul-12	10	9621	5806	8917	4710
15-Jul-12	17	8695	10215	8265	11143
21-Jul-12	23	9647	10254	9665	11301
22-Jul-12	24	10136	10210	9558	10967
27-Jul-12	29	10633	10617	10770	10329
28-Jul-12	30	10391	10858	10901	10581
03-Aug-12	36	12083	10000	11939	10025
04-Aug-12	37	12389	9687	12041	10269
08-Aug-12	41	14065	7154	12978	8472
16-Aug-12	49	13371	6111	12207	5977
25-Aug-12	58	11961	6014	10997	6684
06-Sep-12	70	11165	8783	11582	8126
15-Sep-12	79	11980	5531	11825	7135
21-Sep-12	85	12166	6086	11025	9202
05-Oct-12	99	10715	7615	10510	5679
19-Oct-12	113	11726	7280	10038	6628
25-Nov-12	150	13255	36	12477	2937
13-Dec-12	168	12858	0	13316	305
01-Feb-13	218	9306	0	9421	0
01-Mar-13	246	7464	17	8426	0
09-Mar-13	254	7512	497	8017	660
17-Mar-13	262	6862	1691	7372	2419
23-Mar-13	268	7312	2431	7416	3624
29-Mar-13	274	7720	4097	8018	5165
07-Apr-13	283	9518	7326	9833	8414
13-Apr-13	289	12523	10166	12594	11211
19-Apr-13	295	15677	11324	16221	12975
26-Apr-13	302	20574	9725	20412	14287
03-May-13	309	23935	5808	24100	14521
10-May-13	316	23629	4551	27670	13380
17-May-13	323	16210	6036	30325	12864
26-May-13	332	9981	9778	17904	10157
02-Jun-13	339	9289	7396	26814	3454
14-Jun-13	351	6135	4875	16284	1600
21-Jun-13	358	5590	3089	11501	7725
28-Jun-13	365	4210	1522	10454	10165
08-Jul-13	375	1962	978	11162	7534
26-Jul-13	393	0	0	4817	4498

Note: EXP-2012-A became extinct on July 26 in 2013.

Date	Elapsed day	2013/2014 EXP-2013-A		2013/2014 EXP-2013-B	
		Adult	Brood	Adult	Brood
13-Aug-13	0	7579	4167	5953	4683
24-Aug-13	11	7000	3363	6041	2146
1-Sep-13	19	7324	1825	5565	558
5-Sep-13	23	7487	2261	5549	578
15-Sep-13	33	6415	5462	4027	2665
21-Sep-13	39	6697	4683	3829	3198
27-Sep-13	45	5711	2538	3889	2882
4-Oct-13	52	5048	649	3824	2142
13-Oct-13	61	4210	151	4322	113
27-Oct-13	75	3152	314	3371	0
15-Nov-13	94	2714	50	3181	0
1-Dec-13	110	2189	3	2904	0
5-Jan-14	145	1100	0	2218	0
7-Feb-14	178	0	314	1799	0
28-Feb-14	199	0	0	1594	0

Note: EXP-2013-A became extinct on February 7 in 2014.

Supplementary Table 2. Estimated apparent longevity, *L(t), i* n 2011/2012, 2012/2013and 2013/2014 experiments.

Date	Elapsed Days	2011/2012
		EXP-2011-A
		L(t) [days]
9-Jul-11	0	7.38
16-Jul-11	7	14.85
22-Jul-11	13	14.59
29-Jul-11	20	21.27
6-Aug-11	28	21.89
12-Aug-11	34	22.26
18-Aug-11	40	17.63
26-Aug-11	48	15.17
10-Sep-11	63	18.82
17-Sep-11	70	15.75
24-Sep-11	77	16.44
29-Sep-11	82	16.37
7-Oct-11	90	20.65
21-Oct-11	104	23.79
30-Oct-11	113	26.73
4-Nov-11	118	27.44
18-Nov-11	132	33.75
26-Nov-11	140	39.66
3-Dec-11	147	43.64
17-Dec-11	161	50.76
16-Feb-12	222	107.14
2-Apr-12	268	133.10

Date	Elapsed Days	2012/2013	
		EXP-2012-A	EXP-2012-B
		L(t) [days]	*L(t)* [days]
28-Jun-12	0	19.03	14.49
08-Jul-12	10	25.65	22.15
15-Jul-12	17	21.94	21.07
21-Jul-12	23	21.53	23.06
22-Jul-12	24	21.12	20.76
27-Jul-12	29	14.51	13.24
28-Jul-12	30	13.79	13.15
03-Aug-12	36	15.41	13.52
04-Aug-12	37	14.88	13.70
08-Aug-12	41	16.23	14.86
16-Aug-12	49	18.39	15.60
25-Aug-12	58	19.88	18.37
06-Sep-12	70	23.00	21.68
15-Sep-12	79	21.31	20.47
21-Sep-12	85	21.00	18.04
05-Oct-12	99	21.14	16.13
19-Oct-12	113	19.59	19.03
25-Nov-12	150	47.53	37.74
13-Dec-12	168	64.81	51.75
01-Feb-13	218	108.91	93.76
01-Mar-13	246	133.75	118.44
09-Mar-13	254	141.82	124.67
17-Mar-13	262	148.28	128.57
23-Mar-13	268	154.08	131.34
29-Mar-13	274	159.04	133.66
07-Apr-13	283	166.80	136.13
13-Apr-13	289	172.59	138.63
19-Apr-13	295	177.64	141.88
26-Apr-13	302	181.88	38.93
03-May-13	309	185.32	30.47
10-May-13	316	34.93	28.37
17-May-13	323	26.01	28.76
26-May-13	332	22.57	17.50
02-Jun-13	339	16.76	27.14
14-Jun-13	351	11.18	26.34
21-Jun-13	358	14.36	27.64
28-Jun-13	365	12.24	31.03
08-Jul-13	375	13.31	21.93
26-Jul-13	393	0.00	10.72

Note: EXP-2012-A became extinct on July 26 in 2013.

Date	Elapsed day	2013/2014	
		EXP-2013-A	EXP-2013-B
		L(t) [days]	*L(t)* [days]
13-Aug-13	0	23.02	16.09
24-Aug-13	11	21.26	16.32
1-Sep-13	19	23.77	19.22
5-Sep-13	23	25.14	21.56
15-Sep-13	33	27.73	26.86
21-Sep-13	39	27.08	29.13
27-Sep-13	45	13.68	29.88
4-Oct-13	52	14.39	16.20
13-Oct-13	61	20.18	20.63
27-Oct-13	75	31.34	29.89
15-Nov-13	94	48.37	48.19
1-Dec-13	110	62.34	63.18
5-Jan-14	145	92.61	95.68
7-Feb-14	178	0.00	127.15
28-Feb-14	199		147.03

Note: EXP-2013-A became extinct on February 7 in 2014.

Supplementary Figure 1. Comparison of apparent longevity between previous works (Bühlmann[31], Fukuda & Sekiguchi[2]) and this work.

The mean longevity reported by Fukuda and Sekiguchi (1966) was plotted as apparent longevity as it was. The apparent longevities of Bühlmann (1985) and this work was calculated from the numbers of adult bees and capped (sealed) brood by using our mathematical model on the assumption of the eclosion rate = 0.9. The apparent longevities at the colony extinction ($L(t)$ = 0) were removed when being plotted, which were on July 26 in 2012/2013-A and on February 28 in 2013/2014-A. All data of apparent longevity were plotted from the end of June.

From the figure, we can find that there are following clear distinctions between the previous apparent longevities reported by Fukuda and Sekiguchi (1966) and Bühlmann (1985) and that in this work in which the apparent longevities show very similar season-changes to one another, increasing from early autumn, reaching a maximum at the end of overwintering and falling approximately plumb down after overwintering: (1) The apparent longevity of Fukuda and Sekiguchi (1966) rapidly increases from the end of August and already reaches a maximum (about 140 days) at the beginning of October. Thereafter it begins to decrease gradually and it decreases again in nearly the same decrement from the beginning of April after there is a lack of data from the end of October to the end of March. (2) The apparent longevity of Bühlmann (1985) also rapidly increases from the end of August and reaches a maximum (about 245 days) at the end of February. After it reaches a maximum, it begins to decrease with time from the beginning of March to early May. Thereafter it reaches a certain value (10 – 20 days). That is, after it reaches a maximum, it does not show a sudden drop like our results but do a comparatively gradual decrease during and just after overwintering.

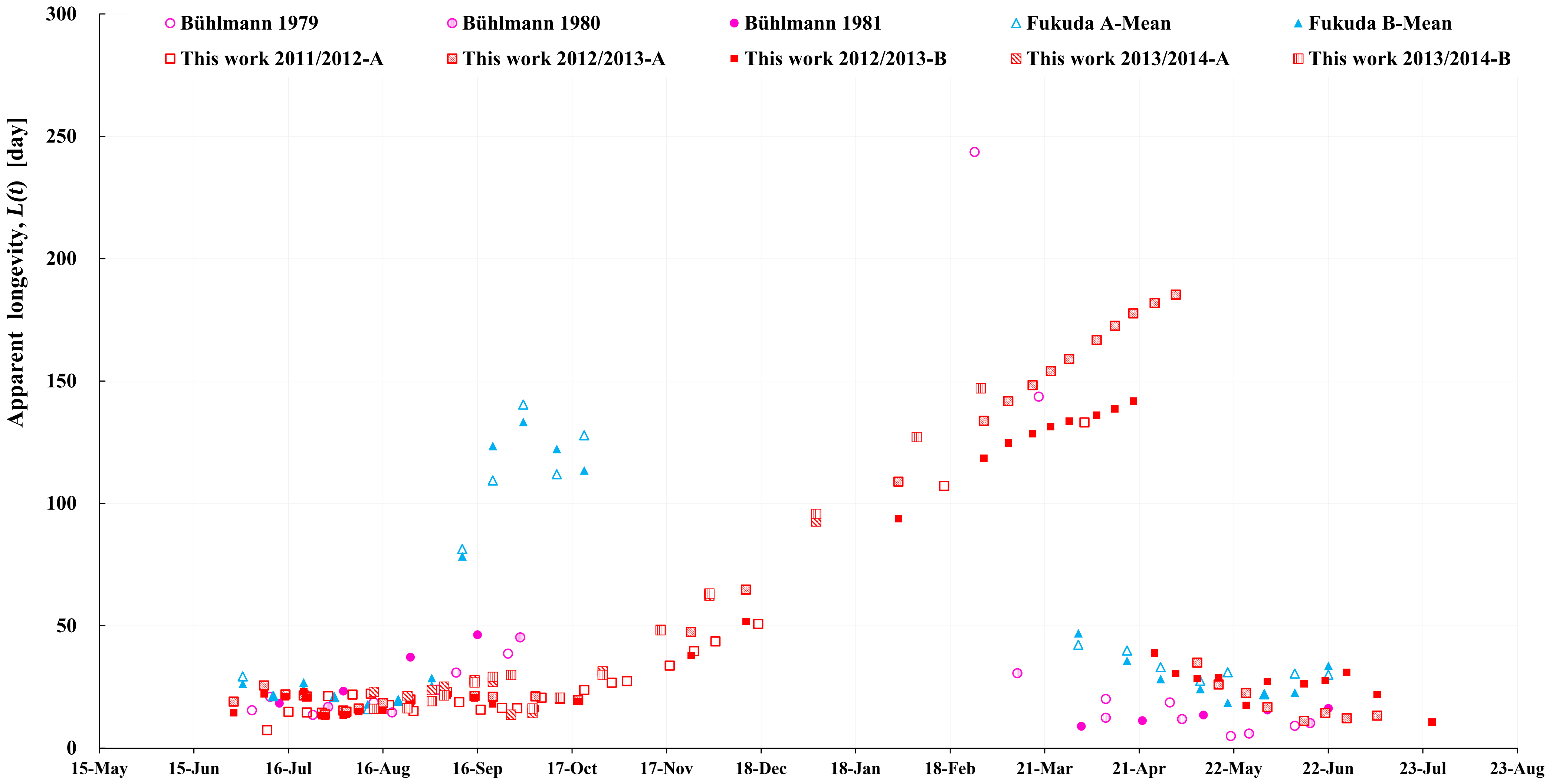

Supplement: Supplementary file 1 — A mathematical model to estimate the seasonal change in apparent longevity of bee colony [file 41598_2019_40725_MOESM1_ESM.pdf]
